# The Value of *NOTCH2NLC* Gene Detection and Skin Biopsy in the Diagnosis of Neuronal Intranuclear Inclusion Disease

**DOI:** 10.3389/fneur.2021.624321

**Published:** 2021-05-04

**Authors:** Jie Pang, Jing Yang, Yanpeng Yuan, Yuan Gao, Changhe Shi, Shiheng Fan, Yuming Xu

**Affiliations:** ^1^Department of Neurology, The First Affiliated Hospital of Zhengzhou University, Zhengzhou, China; ^2^Institute of Neuroscience, Zhengzhou University, Zhengzhou, China; ^3^National Health Commission Key Laboratory of Cerebrovascular Disease, Zhengzhou University, Zhengzhou, China

**Keywords:** gene, leukoencephalopathy, headache, nuclear inclusion bodies, skin biopsy

## Abstract

The clinical manifestations of neuronal intranuclear inclusion disease (NIID) are heterogeneous, and the premortem diagnosis is mainly based on skin biopsy findings. Abnormal GGC repeat expansions in *NOTCH2NLC* was recently identified in familial and sporadic NIID. The comparison of diagnostic value between abnormal GGC repeat expansions of *NOTCH2NLC* and skin biopsy has not been conducted yet. In this study, skin biopsy was performed in 10 suspected adult NIID patients with clinical and imaging manifestations, and GGC repeat size in *NOTCH2NLC* was also screened by repeat primed-PCR and GC-rich PCR. We found that five cases had ubiquitin-immunolabelling intranuclear inclusion bodies by skin biopsy, and all of them were identified with abnormal GGC repeat expansions in *NOTCH2NLC*, among whom four patients showed typical linear hyperintensity at corticomedullary junction on DWI. Five (5/10) NIID patients were diagnosed by combination of *NOTCH2NLC* gene detection, skin biopsy or combination of *NOTCH2NLC*, and typical MRI findings. The diagnostic performance of *NOTCH2NLC* gene detection was highly consistent with that of skin biopsy (*Kappa* = 1). The unexplained headache was firstly reported as a new early phenotype of NIID. These findings indicate that *NOTCH2NLC* gene detection is needed to be a supplement in the diagnose flow of NIID and also may be used as an alternative method to skin biopsy especially in Asian population.

## Introduction

Neuronal intranuclear inclusion disease (NIID) is a rare neurodegenerative disease characterized by the presence of eosinophilic hyaline nuclear inclusion bodies in the cells of the central and peripheral nervous system, internal organs, and skin. The clinical manifestations are extremely heterogeneous such as, dementia, Parkinson's syndrome, peripheral neuropathy, epilepsy, and encephalopathy. It is difficult to diagnose NIID based on clinical manifestations alone. The ante-mortem detection of intranuclear inclusions in NIID cases by skin biopsy was firstly reported in 2011 (1). After that, the number of NIID cases that have been diagnosed with skin biopsy has rapidly increased. Despite of the relative convenience and harmlessness, skin biopsy is undoubtedly invasive for patients with contraindications.

In 2019, researchers from different groups reported GGC repeat expansion of *NOTCH2NLC* gene in familial and sporadic Asian NIID patients, suggesting that *NOTCH2NLC* might be associated with the pathogenesis of the disease (2–5). The advantage of this method was obvious in that genetic detection is more convenient and non-inversion. However, the comparison of diagnostic value between *NOTCH2NLC* gene detection and skin biopsy has not been carried out yet.

In this study, for the first time, the diagnostic consistency between skin biopsy and *NOTCH2NLC* gene detection was analyzed in 10 patients with suspected NIID, in order to explore the diagnostic value of the *NOTCH2NLC* GGC repeat expansion in NIID. The clinical and other imaging characteristics of NIID cases were also studied here.

## Methods

### Patients and Diagnosis

Ten suspected NIID patients with clinical manifestations of NIID as well as bilateral symmetrical white matter lesions (Fazekas score ≥2) at Department of Neurology in the First Affiliated Hospital of Zhengzhou University from June 1, 2019 to June 30, 2020 were included in this study. Those patients with disease-causing gene mutation by whole-exome sequencing were exclued.

This study was approved by the Medical Ethics Committee of the First Affiliated Hospital of Zhengzhou University (2019-KY-294), and all subjects signed an informed consent form.

### Positron Emission Tomography-Computed Tomography and 3.0T MRI

^18^F-FDG PET-CT and 3.0 T-head MRI scans were performed in the MRI Department and PET Center of the First Affiliated Hospital of Zhengzhou University, respectively. MRI scans included T1-weighted sequence, T2-weighted sequence, T2 FLAIR sequence, and DWI.

### Skin Biopsy

Skin biopsies were taken with three-millimeter skin punch at the distal leg (10 cm above the external ankle) and cervical C7 paravertebral area according to the previous study. Skin samples were fixed in 4°C cold Zamboni (G2190, Solarbio) for 24–48 h. The tissues were embedded in paraffin and 4-μm serial sections were sliced. Standard pathological examinations included haematoxylin and eosin (HE) staing, anti-ubiquitin antibody (ab7780, Abcam, UK) immunostaining (6). The presence of eosinophilic or ubiquitin-positive inclusion bodies in the nuclei of sweat gland cells, fibroblasts, and fat cells was observed at 100 × magnification under light microscope (Ni-U, Nikon, Japan). One-third of each skin sample was cut for transmission electron microscopy and fixed in 2.5% glutaraldehyde solution with phosphate buffer (pH 7.3). Images were captured under an electron microscope (JEM-1400; JEOL, Japan).

### Analysis of *NOTCH2NLC* GGC Repeats

Genomic DNA was obtained from the venous blood of all participants using standard methods (Tiangen Gene Extraction Kit, TIANGEN Biotech (Beijing) Co., Ltd., Beijing, China). Based on previously reported methodology (4, 5), repeat primed polymerase chain reaction (RP-PCR) and GC-rich PCR analysis were used to detect the presence of repetitive sequences in the GGC fragment of *NOTCH2NLC* and determine the exact number of repetitive sequences. In RP-PCR, fluorescein (FAM)-labeled gene-specific primers (5′-CCTCAGCCCGATACTCACCAT-3′) and primers containing repetitive sequences (5′-TACCAATACGCATCCCGCGATTTGTCTTA(CGG)5-3′) were used to identify the amplification of CGG repetitive sequences. In GC-PCR, FAM was used to label the forward (5′-CTGACCTTTCAAGATCCTGCTTTCATCCCAGCT-3′) and reverse (5′-AAGTGCCTTACTTTGCGTAGCTGTGTGCTTGGCAGT-3′) primers to determine the repeat size. After 10 min of incubation at 98°C, the following cycle conditions were performed: 16 cycles at 98°C for 30 s, 66°C for 1 min, with each cycle lowered by 0.5°C, and at 68°C for 8 min; thereafter, 32 cycles were performed at 98°C for 30 s, 58°C for 1 min, and 68°C for 8 min; and finally, an extension step at 68°C for 10 min. A 3,500 × L Genetic Analyzer (Applied Biosystems, Foster City, CA, USA) was used to perform capillary electrophoresis of the PCR products to analyse the fragment length; GeneScan 1,000 ROX Size Standard (Applied Biosystems) was used to determine the allele size.

### Statistical Analysis

SPSS 21.0 statistical software was used for statistical analysis. Normally distributed data are represented as mean ± standard deviation ($\bar{\text{x}}$ ± s) or median. Categorical variables are represented by rate, and count data are represented by the number of cases and fractions. Comparisons between groups were tested by Fisher's exact probability method and the consistency with the Kappa consistency test. *P* ≤ 0.05 indicated statistically significant results.

## Results

Among the 10 suspected adult NIID patients, five cases were diognosed with NIID by combination of NOT*CH2NLC* GGC repeat detection, skin biopsy, and typical MRI imagings. Details of the five NIID patients are summarized in Table 1.

**Table 1:** Demographic and clinical data of patients with NIID.

	**1**	**2**	**3**	**4**	**5**
**Age of onset (years)**	52	62	76	46	48
**Family history**	–	+	–	–	+
**Clinical manifestations**					
Dementia	–	+	+	+	–
Parkinson's syndrome	–	+	–	–	–
Peripheral neuropathy	+	–	+	+	–
Muscle weakness	–	–	+	+	–
Sensory disturbance	+	–	+	+	–
Autonomic symptoms	+	+	+	+	+
Diarrhea	–	+	–	–	–
Constipation	–	–	+	–	–
Abnormal sweating	+	+	+	+	–
Orthostatic dizziness	+	–	+	–	–
Abnormal pupil contraction	–	–	++	+	+
Disturbance of consciousness	–	–	+	–	–
Headache	+	+	+	–	+
**Head MRI**					
High linear signals on DWI	–	+	+	+	+
Leukoencephalopathy	+	+	+	+	+
Fazekas score	2	2	3	3	2
Ventricular distension	–	+	+	+	+
Pons hyperintensity	–	–	+	–	+
^**18**^**F-FDG PET-CT**					
Decreased glucose metabolism	NA	+	NA	NA	NA
**Skin biopsy**					
HE staining	+	+	+	+	+
Ubiquitin immunostaining	+	+	+	+	+
Electron microscopy	+	–	+	NA	+
***FMRI*** **gene CGG repeats**	30	30	31	25	33
***NOTCH2NLC*** **gene GGC repeats**	66	128	105	103	142
**Cognitive function tests**					
MMSE	28	16	2	20	28
MoCA	22	13	0	19	NA
**Laboratory data**
CSF cell (>5/mm^3^)	+	NA	NA	NA	+
CSF Glucose (<50 mg/dL)	+	NA	NA	NA	+
CSF protein (>45 mg/dL)	+	NA	NA	NA	+

“*+”, yes or positive; “–”, none or negative; NA, data not available; MMSE, Mini-Mental State Examination; MoCA, Montreal Cognitive Assessment Scale*.

### Clinical Manifestation

Among the five patients with confirmed NIID, two were male and three were female, all aged 48–76 (58.4 ± 10.31) years, with the age of NIID onset at 48–76 (58.4±10.12) years. Three patients had acute or subacute onset, and two had chronic onset. The course of the disease was 0.3–24 (5.96 ± 10.15) months, with a median of 2 months.

Two patients had a family history (2/5, 40%) of disease, and three were sporadic (3/5, 60%). There was no obvious difference between the familial and sporadic types in clinical manifestations, and the heterogeneity of manifestations was high. Autonomic dysfunction was the most common manifestation (5/5, 100%), abnormal sweating was the most common type of autonomic dysfunction (4/5, 80%), followed by abnormal pupil contraction (3/5, 60%), gastrointestinal symptoms (2/5, 40%), postural dizziness (2/5, 40%), and abnormal bladder function (1/5, 20%). The second most common manifestation was headaches (4/5, 80%), dementia (3/5, 60%), and peripheral neuropathy (3/5, 60%). In addition, other clinical manifestations such as, muscle weakness (2/5, 40%), Parkinson's syndrome (1/5, 20%), and disturbance of consciousness (1/5, 20%) were observed.

### Head MRI and PET-CT

**Figure 1:**
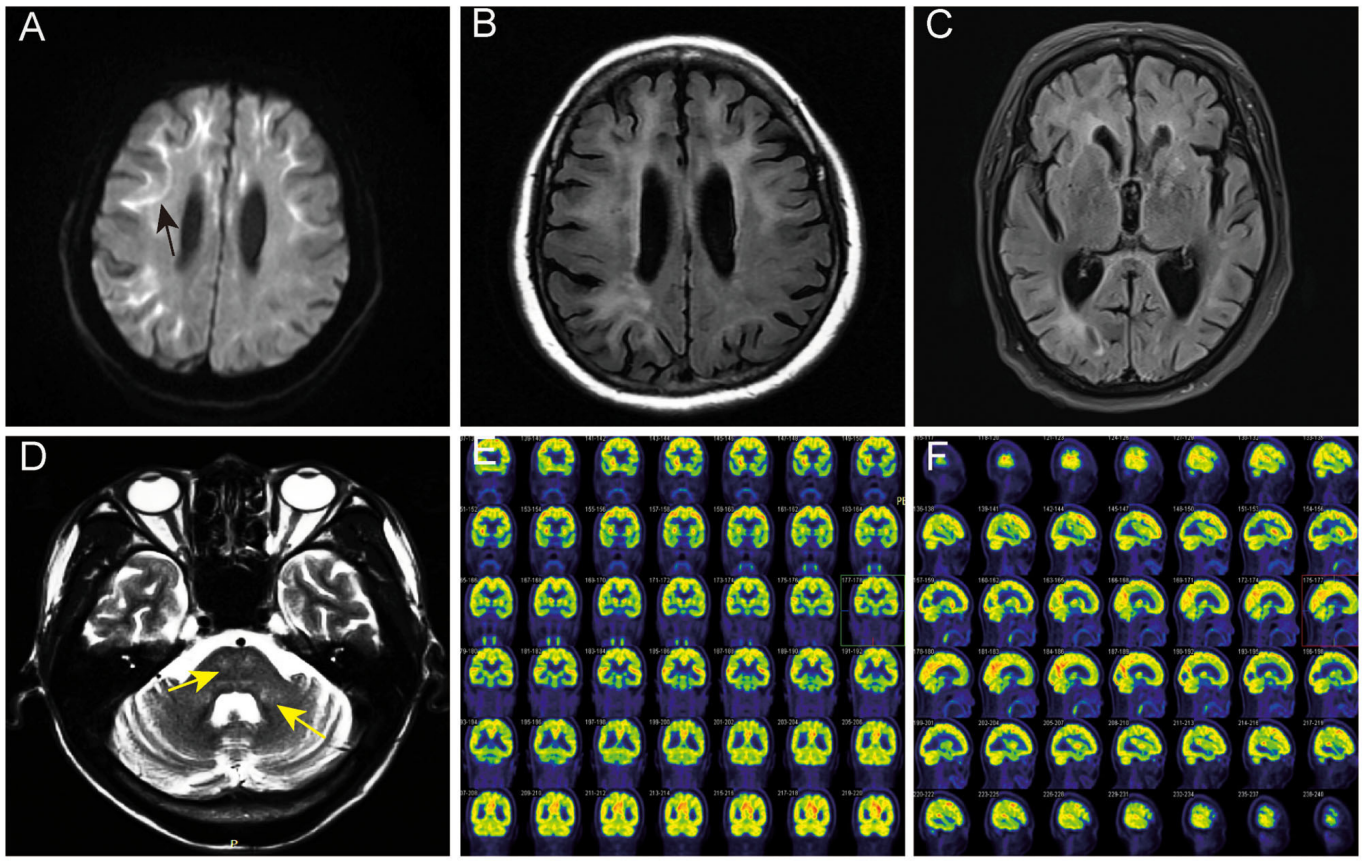
Head MRI and PET-CT findings of NIID cases. **(A)** The linear high signals on DWI of subject 2 in the corticomedullary junction (black arrow). **(B)** Bilateral paraventricular white matter hyperintensity on T2 FLAIR of subject 2. **(C)** Ventricular dilation on T2 of subject 2. **(D)** Hyperintensity areas in the cerebral pontine and the middle cerebellar peduncles on T2 FLAIR (yellow arrow). **(E,F)** The^18^F-FDG PET-CT of subject 2 showed decreased metabolism in the left frontal lobe and right parietal lobe.

Among the five patients with NIID, four showed typical high-intensity signals on DWI in the corticomedullary junction (4/5, 80%) (Figure 1A), five showed bilateral paraventricular white matter hyperintensity on T2 FLAIR (5/5, 100%) (Figure 1B), four showed enlarged ventricles (4/5, 80%) (Figure 1C), two showed T2 FLAIR hyperintensity areas in cerebral pontine (2/5, 40%) (Figure 1D), and one showed T2 FLAIR hyperintensity areas in the middle cerebellar peduncles (MCP sign) (1/5, 20%) (Figure 1D).

One case of NIID showed decreased glucose metabolism in the left frontal lobe, right parietal lobe, and bilateral ventricular anterior and posterior horns upon ^18^F-FDG PET-CT (Figures 1E,F), which is consistent with the high signal range of corticomedullary junction on DWI.

### Skin Biopsy Findings and GGC Repeat Expansions in *NOTCH2NLC*

**Figure 2:**
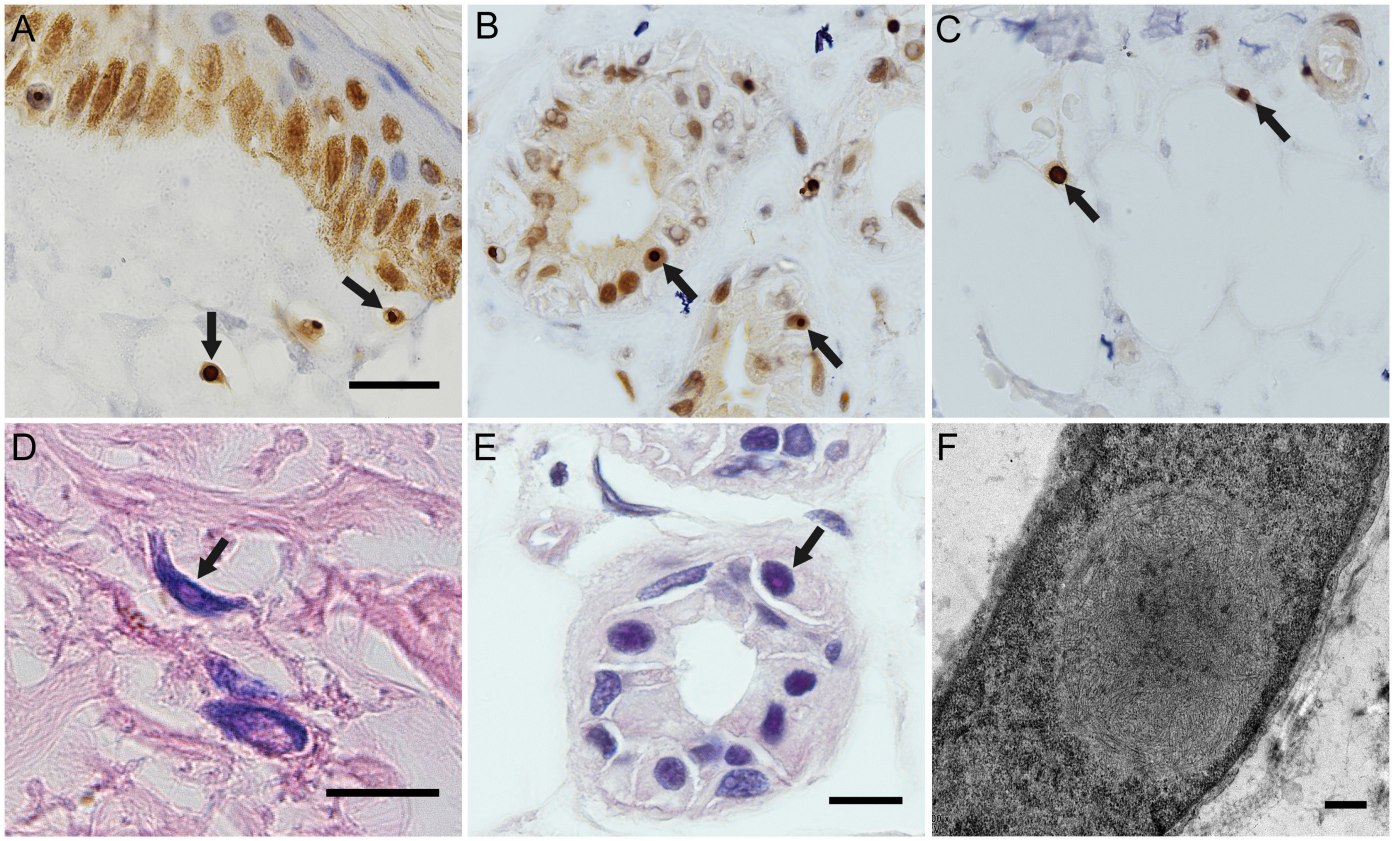
Histopathological findings of NIID cases. Ubiquitin immunostaing inclusion bodies in the nuclei of the fibroblasts of subject 4 **(A)**, of the sweat gland cells of subject 5 **(B)**, and of the fat cells of subject 5 **(C)**. Eosinophilic intranuclear inclusion bodies in the fibroblasts of subject 2 **(D)** and in the sweat gland cells of subject 5 **(E)**. Intranuclear inclusion bodies in the fibroblasts of subject 5 under electron microscope **(F)**. Arrows indicated intranuclear inclusion bodies. Scale bars: **(A–E)** 10 μm; **(F)** 500 nm.

The eosinophilic and ubiquitin-positive nuclear inclusion bodies were found in the fibroblasts (Figures 2A,D), sweat gland cells (Figures 2B,E), and fat cells (Figure 2C). The 10-nm diameter intranuclear inclusions were also found in fibroblasts (Figure 2F) and sweat gland cells under eletron microscopy. Among the five cases of confirmed NIID, the positive rate of cutaneous eosinophilic inclusion bodies was 100% in HE-staining (5/5), 100% with ubiquitin labeling (5/5), and 60% under electron microscopy (3/5).

**Figure 3:**
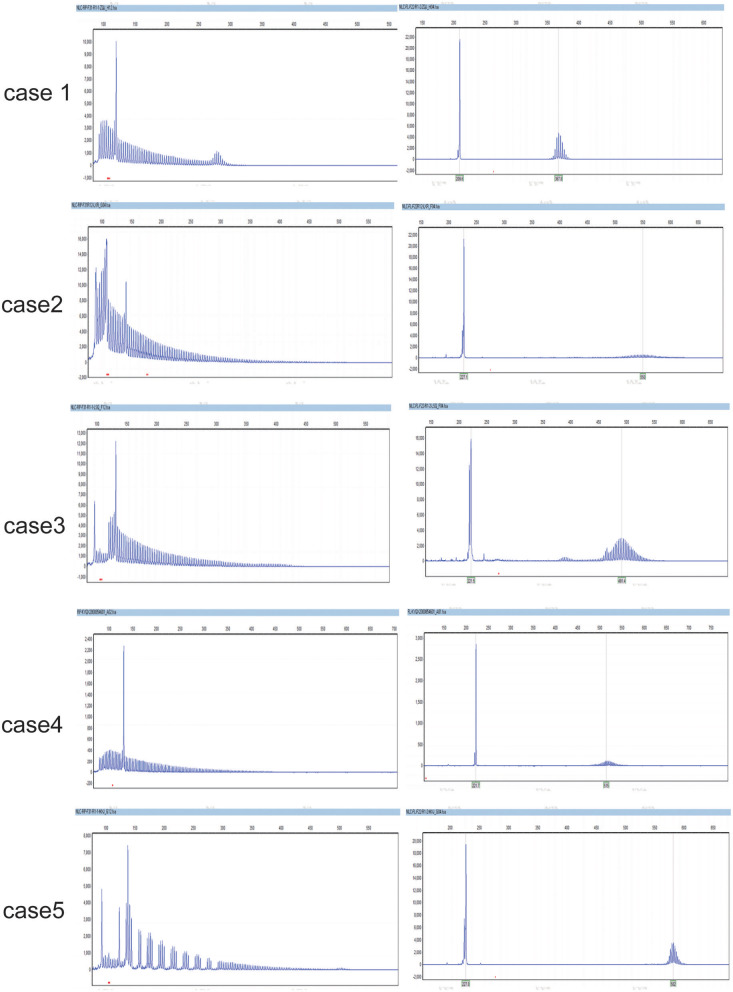
The GGC repeats in the two alles of *NOTCH2NLC* in NIID cases. They were respectively, 66,12 (case 1); 128,20 (case 2); 105,15 (case 3); 103,20 (case 4); 142,18 (case 5).

Expanded GGC repeats were found in one allele of *NOTCH2NLC* in each patient, they were, respectively 66, 128, 142, 105, and 103 (Figure 3).

The sensitivity, specificity, the positive predictive value, and the negative predictive value of the skin biopsy or *NOTCH2NLC* GGC repeat expansions was all 5/5 (100%), respectively.

### Diagnostic Consistency of Skin Biopsy and *NOTCH2NLC* GGC Repeat Detection

The diagnostic consistency of skin biopsy and *NOTCH2NLC* GGC repeat detection was compared by Fisher's exact probability method and Kappa consistency analysis. The *NOTCH2NLC* GGC repeat expansions were completely consistent with skin biopsy (*P* = 0.008, K = 1).

## Discussion

The main findings of the present study included that (1) Five NIID cases among the 10 suspected NIID patients were diagnosed by combination of *NOTCH2NLC* GGC repeat detection, skin biopsy and typical DWI images; (2) The diagnostic efficiency of *NOTCH2NLC* GGC repeat expansions detection was completely consistent with skin biopsy; (3) The unexplained headache was reported as an early phenotype of NIID. As to NIID, we proposed firstly that the *NOTCH2NLC* GGC repeat expansions was highly consistent with skin biopsy findings, *NOTCH2NLC* gene detection may be a supplement in the diagnose of NIID, and may also be used as an alternative method of skin biopsy.

Before the Sone's diagnostic criteria came out, the diagnosis of NIID mainly depended on autopsy, and the number of cases reported in the world was very small. After 2011, Sone first found ubiquitin positive inclusion bodies in skin biopsies of patients with NIID, while no inclusion bodies were found in other diseases with neural inclusion bodies. The inclusion bodies in the skin biopsies were consistent with the intracranial lesions (1). After the report of Sone diagnostic criteria in 2016 (7), a series of NIID cases were found, with the most common cases in Asia. By 2020, most of the reported cases were confirmed by skin biopsy (8–10). Most of these cases were diagnosed with Sone's 2016 diagnostic criteria, including positive skin biopsy, typical MRI findings, and negative FMR1 GGC expansion (7).

According to Sone's 2016 diagnostic criteria, a typical DWI linear high signal at the corticomedullary junction is a necessary condition for the diagnosis of NIID, unless it is accompanied by decreased nerve conduction velocity, miosis or increased cerebrospinal fluid protein. In the present study, one NIID patient did not show the typical DWI feature, but she had symmetrical paraventricular white matter hyperintensity on T2 FLAIR image, and intranuclear inclusion bodies in the skin and expanded GGC repeats of 66 in *NOTCH2NLC* gene. This result demonstrated that the characteristic DWI image did not necessarily occur in early stage of NIID. This result is similar to that of Okubo et al. (11), among 12 NIID patients with leukoencephalopathy, they found that three early patients did not have typical DWI findings, suggesting that typical DWI findings often appear before non-specific MRI findings, such as, leukoencephalopathy.

In 2019, the Chinese and Japanese researchers reported non-coding GGC repeat expansions in *NOTCH2NLC* (also named NBPF19) gene in familial and sporadic NIID patients (3–5). At present, most researchers believe that GGC repeat expansions in *NOTCH2NLC* as the causative mutations for NIID. In previously reported 143 NIID patients who underwent skin biopsy and *NOTCH2NLC* gene detection at the same time, we found that there was a high degree of consistency between skin biopsy and *NOTCH2NLC* gene detection in the remaining patients, except for one patient whose skin biopsy was negative and was carrier of an expansion of NOTCH2NLC GGC repeats (12). Recent studies have shown that abnormal GGC amplification in *NOTCH2NLC* can also occur in Parkinson's disease (PD), Alzheimer's disease (AD), unexplained white matter disease, multiple system atrophy (MSA), and essential tremor (ET) (5, 11, 13, 14). Skin biopsies were performed in a few of these patients who completed *NOTCH2NLC* gene detection and found positive for ubiquitin related antibodies. Therefore, it suggests that the GGC repeat expansion of *NOTCH2NLC* gene is consistent with skin ubiquitin positive inclusion bodies in both NIID and other neurodegenerative diseases. Of course, all of these patients with skin biopsy were alive without autopsy diagnosis. The other neurodegenerative diseases such as AD, PD, MSA, or ET, with *NOTCH2NLC* GGC expansions and skin intranuclear inclusions may be NIID in nature. One most recent study showed that 3/13 sporadic PD patients had abnormal *NOTCH2NLC* GGC repetition but did not demonstrate clinical or imaging manifestations of NIID over 9-year follow-ups (15). Unfortunately, none of the three PD patients underwent skin biopsy or autopsy to determine the presence of neuronal intranuclear inclusions. Therefore, the three PD patients were only clinically diagnosed PD, and the possibility of NIID could not be completely ruled out. The results of our study showed that GGC repeat expansions in *NOTCH2NLC* gene showed almost similar diagnostic consistency with skin biopsy as we expected. In conclusion, we suggest that *NOTCH2NLC* gene detection is highly consistent with skin biopsy, and even surpasses skin biopsy to some extent. We suggest that *NOTCH2NLC* gene testing should be added to the NIID diagnostic process, or even replace skin biopsy.

In 2020, a new European study reported that no abnormal *NOTCH2NLC* GGC expansion was found in 11 NIID patients confirmed by autopsy, but none of these patients underwent skin biopsy (16). In this study, the number of GGC repeats of *NOTCH2NLC* gene in one patient with positive skin biopsy was 58, which met the standard of intermediate-length repeat expansions reported by us (17). Therefore, it should be noted that the GGC abnormal expansion of the *NOTCH2NLC* gene may not be the only one causative gene of NIID, especially for NIID patients in the European population. Surely, studies with larger samples are still needed to confirm this conclusion.

In this study, we also noted that headache, a common clinical symptom, was also common in patients with NIID. Among our patients, two patients even consulted the doctor with headache as the earliest and main clinical symptom. Hemiplegic migraine was once reported as rare clinical manifestations in 2019, no other types of headache were reported yet (18–20). However, the previous literature rarely included headache among the common symptoms of NIID. It is suggested that during the diagnosis of headache, we should inquire about other nervous system symptoms and signs such as, autonomic nerve disorders, dementia, myasthenia and so on. If necessary, we should improve the head MRI, skin biopsy, or *NOTCH2NLC* gene detection. NIID should be considered as one cause of headache. We also performed ^18^F-FDG PET-CT scan on one patient with NIID. This patient's ^18^F-FDG PET-CT results showed decreased metabolism in the left frontal lobe, right parietal lobe, and bilateral ventricular anterior and posterior horns, and these results were consistent with the high signal range on DWI of her previous MRI and the only one PET study on NIID patient in 2019 (21).

There are several limitations in this study. Firstly, we studied in a small portion of NIID cases without OPDM, OPML, or FXTAS cases in a single center. This is a pilot study, a larger sample study from multi-centers is already going on, which may produce more solid results in relation with skin biopsy and NOTCH2NLC genetic testing. Secondly, there may be a certain selection bias since the suspected NIID patients were selected from those with severe leukoencephalopathy, so the conclusion is more likely to be applicable to NIID patients with leukoencephalopathy. Even so, this is a preliminary conclusion, the definite conclusion could be finally made until post-mortem dissection was done.

To sum up, we preliminary suggest that *NOTCH2NLC* gene detection is needed to be a supplement in the diagnose of NIID and it can improve the diagnostic reliability of NIID by the combination of characteristic MRI findings and skin biopsy, especially when there is no characteristic manifestation on DWI. We also propose that *NOTCH2NLC* gene detection may be used as an alternative diagnostic method of skin biopsy for pre-mortem NIID diagnosis, especially in Asian population.

## Data Availability Statement

The datasets presented in this study can be found in online repositories. The names of the repository/repositories and accession number(s) can be found in the article/supplementary material.

## Ethics Statement

The studies involving human participants were reviewed and approved by the Medical Ethics Committee of the First Affiliated Hospital of Zhengzhou University (batch number: 2019-KY-294). The patients/participants provided their written informed consent to participate in this study.

## Author Contributions

All authors listed have made a substantial, direct and intellectual contribution to the work, and approved it for publication.

## Conflict of Interest

The authors declare that the research was conducted in the absence of any commercial or financial relationships that could be construed as a potential conflict of interest.
